# Prevalence, outcomes, costs, and treatments of a contemporary population with chronic kidney disease in Norway: a nationwide observational study

**DOI:** 10.1186/s12882-025-04171-7

**Published:** 2025-07-17

**Authors:** Trond Geir Jenssen, Johan Bodegård, Kari Anne Sveen, Marcus Thuresson, Kåre I. Birkeland

**Affiliations:** 1 Nephrology Section, Department of Transplantation Medicine, Oslo University Hospital, Oslo, Norway; 2 Institute of Clinical Medicine, University of Oslo, Oslo, Norway; 3 RWE Research AS, Oslo, Norway; 4 Statisticon AB, Uppsala, Sweden

**Keywords:** Chronic kidney disease, Type 2 diabetes, Epidemiology, Hospital health care costs, Sodium-glucose cotransport-2 inhibitors, Renin-angiotensin system inhibitors

## Abstract

**Background:**

This nation-wide study describes patients with diagnosed chronic kidney disease (CKD), with and without type 2 diabetes (T2D).

**Methods:**

Prevalence, key adverse outcomes, health care costs, and use of kidney-protective treatment, up until December 31st, 2022, were described in patients aged > 18 years in Norway using register-based data. Only diagnosis codes were used to identify patients with CKD, with laboratory measurements of estimated-glomerular filtration rate and albuminuria unavailable. Utilisation of sodium-glucose cotransporter-2 (SGLT-2) inhibitors and renin-angiotensin system [RAS] inhibitors were evaluated in new users following the first Norwegian approval of an SGLT-2 inhibitor for CKD treatment.

**Results:**

Approximately 3% (125,163 patients) of adults in Norway had diagnosed CKD (average age 70 years, 42% women, 73% without T2D). When describing patients with or without T2D, history of heart failure (22% *versus* 22%), atherosclerotic cardiovascular disease (ASCVD; 57% *versus* 51%), and atrial fibrillation (24% *versus* 27%) were similar. Larger proportions of those with T2D received SGLT-2 inhibitors (24% *versus* 4%) and/or RAS inhibitors (63% *versus* 47%). Hospitalisations for CKD (28.1 *versus* 22.1 events per 100 patient years), heart failure (12.6 *versus* 9.8), myocardial infarction (3.9 *versus* 2.2), and stroke (3.2 *versus* 2.3) were more common in patients with CKD and T2D than those without T2D. However, mortality (10.8 *versus* 8.5) was higher in patients without T2D. CKD and heart failure costs were higher than those for ASCVD, and generally higher in patients with T2D. SGLT-2 inhibitor utilisation increased two-fold the year after its approval but was still low, used mostly at its highest target dose. Discontinuation rates were lower with SGLT-2 inhibitors than with RAS inhibitors, the latter mostly utilised at low doses.

**Conclusions:**

A CKD diagnosis was associated with substantial morbidity and mortality, costs, and undertreatment, both in patients with and without T2D. Use of novel kidney-protective treatment has increased, but an urgent need to improve the utilisation of kidney-protective medications remains, particularly in patients without T2D.

**Clinical trial number:**

Not applicable.

**Supplementary Information:**

The online version contains supplementary material available at 10.1186/s12882-025-04171-7.

## Background

Chronic kidney disease (CKD) and type 2 diabetes (T2D) are two of the most prevalent noncommunicable diseases globally, often coexisting. Estimated to affect one in ten people worldwide, the prevalence and burden of CKD are associated with increased morbidity and mortality, and are expected to increase as the population ages [1–5].

Recent treatment guidelines for CKD and its risk factors include sodium-glucose cotransporter-2 (SGLT-2) inhibitors [6, 7], which have demonstrated beneficial clinical efficacy on kidney disease progression and mortality, irrespective of diabetes status [1, 8]. Until now, two SGLT-2 inhibitors have been approved in Europe for use in patients with CKD, dapagliflozin being the first [3]. While observational studies have described the risks and costs of CKD [2, 3, 5], the rapid change in its management with such kidney-protective medications establishes a continuous need to understand the burden of CKD in a real-world contemporary setting.

Using nationwide healthcare registries in Norway, the aim of this study was to estimate prevalence, key adverse outcomes, health care costs, and use of kidney-protective treatment in patients with diagnosed CKD, with a focus on those with or without T2D. Additionally, this study aimed to describe utilisation of the first SGLT-2 inhibitor approved for CKD treatment, dapagliflozin, and renin-angiotensin system [RAS] inhibitors following new initiation with these kidney-protective treatments.

## Materials and methods

### Data sources

The study was set in Norway, which has a comprehensive, nationwide, public healthcare system that residents can access with a minor co-payment for healthcare visits, hospitalisations, and the filling of drug prescriptions. In any such contact with the healthcare system, it is mandatory that the residents of Norway provide their unique personal identification number, providing a basis for complete population-wide medical history.

Data were extracted from Norway’s Patient Register, Prescription Database, and Cause of Death Register (Supplemental Methods 1) and were linked by the Norwegian Institute of Public Health using the patient’s personal identification number. The linked database was managed by Statisticon AB (Uppsala, Sweden).

The Regional Ethics Committee, *Helse Sør-Øst*, waived the need for informed consent from the patients included in the study, given that the study only used data that had already been collected.

### Patients

This nationwide, observational study included patients aged > 18 years with diagnosed CKD, defined as registered diagnoses of chronic, acute on chronic, hypertensive, diabetic, glomerular, and/or renal-tubulo-interstitial kidney diseases, and/or record of dialysis [3, 5], in the Norwegian Patient Register (Supplemental Methods 2). Measures of estimated-glomerular filtration rate and urine albumin-to-creatinine ratio were not available to identify patients with CKD. Four cohorts were defined to describe the following: *Cohort 1*, the most contemporary patient characteristics; *Cohort 2*, one-year event rates; *Cohort 3*, five-year hospital health care costs; and *Cohort 4*, patterns of treatment with SGLT-2 inhibitors and RAS inhibitors. Patients could have been included in more than one cohort and were grouped according to T2D status; with or without T2D (Supplemental Methods 3). Use of non-steroid mineral corticoid antagonists as nephroprotective treatment was not reimbursed by the authorities during the study period.

### Patient characteristics

The characteristics of patients with diagnosed CKD were evaluated in *Cohort 1*, which included those who were alive as of December 31st, 2022. All diagnoses in any position in outpatient care and/or in inpatient care recorded on and prior to that index date for characterisation were used to identify comorbidities (Supplemental Methods 4). Use of medications were searched for during the year prior to the index date, on and between January 1st, 2022, and December 31st, 2022 (Supplemental Methods 5). Differences in characteristics between patients with or without T2D were evaluated using standardised differences, with values > 10% indicating non-negligible differences.

### Adverse outcomes

Key adverse outcomes including hospitalisations for CKD, heart failure, myocardial infarction, stroke, or peripheral artery disease, and all-cause, cardiovascular, or renal death were monitored in *Cohort 2*, which included patients with diagnosed CKD indexed December 31st, 2021. Patients were followed from that date until December 31st, 2022, or their death, whichever occurred first. Analyses were performed using broad and strict outcome definitions: Broad definition, diagnoses in any position in inpatient care; Strict definition, only diagnoses in the main position in outpatient and/or inpatient care. Adverse outcomes were presented as the one-year event rate per 100 patient years based on the time to the first event. The analysis of event rates is descriptive, and between-group statistical comparisons were not performed.

### Hospital health care costs

Hospital health care costs were calculated for *Cohort 3*, which included patients with diagnosed CKD indexed December 31st, 2017. Costs were extracted from data containing the actual visit costs as charged by the health care provider (i.e., the cost reflects the true reimbursement claim to the local payer). These costs were cumulatively summated during the five years after index and, importantly, includes costs for all first and repeated events, during follow-up, associated with the key adverse outcomes described above. Costs were censored from calculations upon death.

All adverse outcomes were analysed independently from each other and, thus, hospitalisations where more than one adverse outcome was diagnosed contribute to the costs of each included diagnosis. Hence, the costs of two diagnoses could not be added together to form a combined cost. Costs were expressed in Norwegian Kronor and Euros, where one Norwegian Kronor equated to 0.099 euros; the average conversion rate over the five-year follow-up period. The analysis of hospital health care costs are descriptive and formal between-group comparisons were not performed.

### The uptake of SGLT-2 inhibitors

Uptake of the SGLT-2 inhibitor, dapagliflozin, before and after its approval for CKD treatment in Norway (December 21st, 2021) was assessed daily on and between January 1st, 2020, and December 31st, 2022. The proportion of patients with CKD who used dapagliflozin each day was calculated by dividing the total number of patients with a supply of dapagliflozin covering the date of interest divided by the total number of patients with CKD who were alive on that day. The duration of a medication supply was calculated for each filled prescription by dividing the number of pills dispensed by an assumed prescribed dose of one pill per day.

### Utilisation of kidney-protective medications

Utilisation of the SGLT-2 inhibitor, dapagliflozin, and RAS inhibitors was assessed in *Cohort 4*, which included new users of either medication following the approval of dapagliflozin in Norway (December 21st, 2021). Index was the date that dapagliflozin or RAS inhibitor treatment was first initiated in patients previously naïve to the respective medications. Patients were followed for 12 months from index or up until December 31st, 2022, or their death, whichever occurred first.

Given that dapagliflozin has a guideline-recommended target dose of 10 mg [3], the initiation of dapagliflozin treatment was defined as the first-recorded filled prescription of the medication at that dose. Any reduction in dose to 5 mg, which dapagliflozin can also be used at, was tracked thereafter. Hence, dapagliflozin was categorized into two dose levels throughout follow-up: a target dose, 10 mg, and an intermediate dose, 5 mg [3].

Using the highest dose available in Norway according to registered drug prescription doses (Supplemental Methods 6), the RAS inhibitors analysed in this study were categorized into three dose levels: low (< 50% of the highest dose available), intermediate (50–99% of the highest dose), and high (100% of the highest dose) [3].

The duration of a medication supply was calculated as above. If it were calculated that a patient’s medication supply had finished, the patient was considered to have discontinued treatment until a new prescription was filled. Thus, persistence is based on the proportion of patients who had a medication supply on any given day [3].

## Results

### Prevalence and characteristics

At the end of 2022, 3% of the adult population of Norway (125,163 patients among 4,247,835 adults) were estimated to have diagnosed CKD (average age, 70 years; 42% women; 73% without T2D). When describing patients with and patients without T2D, the proportions with a history of heart failure (22% *versus* 22%, respectively), atherosclerotic cardiovascular disease (ASCVD: myocardial infarction, stroke, and/or peripheral artery disease; 57% *versus* 51%), and atrial fibrillation (24% *versus* 27%) were similar (Table 1). Compared to patients without T2D, a larger proportion of those with T2D received kidney-protective treatment (SGLT-2 inhibitors, 24% *versus* 4%; RAS inhibitors, 63% *versus* 47%).

**Table 1:** The characteristics of patients in Norway with a registered diagnosis of chronic kidney disease as of December 31st, 2022

	All patients with CKD*	CKD with T2D	CKD without T2D	Standardised difference (%)
**n**	125,163	32,526	91,456	
**Age**,** years (SD)**	70 (16)	70 (14)	70 (17)	6.6
**Female**,** n (%)**	51,981 (42)	13,049 (40)	38,371 (42)	3.7
**CKD stages and types**,** n (%)**				
Chronic	58,183 (51)	14,021 (53)	43,819 (51)	5.8
CKD stage recorded	48,755 (39)	12,231 (38)	36,205 (40)	
Stage 1–2	6,916 (14)	1,192 (10)	5,684 (16)	17.9
Stage 3–4	34,365 (70)	9,015 (74)	25,177 (70)	9.3
Stage 5	7,474 (15)	2,024 (17)	5,344 (15)	4.9
Hypertensive	6,633 (6)	1,689 (6)	4,906 (6)	3.3
Glomerular diseases	8,771 (7)	2,244 (7)	6,371 (7)	0.3
Renal tubulo-interstitial diseases	28,230 (23)	9,884 (30)	18,076 (20)	24.7
Dialysis	6,270 (6)	1,589 (6)	4,594 (5)	3.3
**Comorbidities**,** n (%)**				
Heart failure	26,929 (22)	6,994 (22)	19,839 (22)	0.5
Atherosclerotic cardiovascular disease	65,613 (52)	18,407 (57)	46,855 (51)	10.8
Myocardial infarction	22,666 (18)	7,254 (22)	15,274 (17)	14.2
Stroke	14,107 (11)	3,930 (12)	10,110 (11)	3.2
Peripheral artery disease	11,641 (9)	3,879 (12)	7,633 (8)	11.9
Atrial Fibrillation	32,176 (26)	7,803 (24)	24,307 (27)	6.0
Severe hyperkalemia	8,434 (7)	2,655 (8)	5,675 (6)	7.6
Cancer	38,535 (31)	9,243 (28)	29,186 (32)	7.6
**Medications**,** n (%)**				
SGLT2 inhibitors	11,442 (9)	7,738 (24)	3,666 (4)	59.7
RAS inhibitors	64,036 (51)	20,463 (63)	42,941 (47)	32.5
MRA	87,56 (7)	2,707 (8)	6,000 (7)	6.7
Statins	61,977 (50)	22,742 (70)	38,525 (42)	58.3
Loop-diuretics	30,031 (24)	9,671 (30)	20,153 (22)	17.6
Low-dose aspirin	35,851 (29)	12,292 (38)	23,251 (25)	26.8
Potassium binders	2,288 (2)	756 (2)	1,491 (2)	5.0
**Diabetes medication**,** n (%)**				
Metformin	15,386 (12)	15,334 (47)	0 (0)	133.6
Sulphonylureas	2,071 (2)	2,065 (6)	0 (0)	36.8
DPP-4 inhibitors	7,068 (6)	7,054 (22)	0 (0)	74.4
GLP-1 receptor agonists	11,145 (9)	11,065 (34)	0 (0)	101.5
Insulin	15,889 (13)	14,723 (45)	0 (0)	128.6

*The total population with CKD characterized in the table includes patients with type 1 diabetes. CKD denotes chronic kidney disease; T2D, type 2 diabetes; SD, standard deviation; SGLT2, sodium glucose transport-2; RAS, renin-angiotensin system; MRA, mineral corticoid antagonists; DPP-4, Dipeptidyl peptidase-4; GLP-1, glucagon-like peptide-1. Standardised differences > 10% indicate non-negligible differences

### Outcome risks

**Fig. 1:**
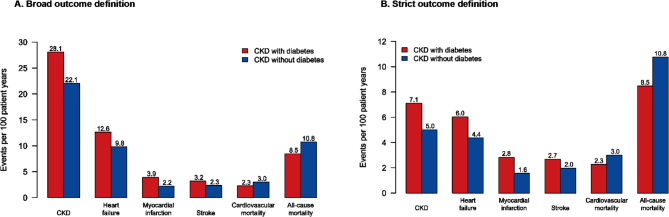
One-year event rates of hospitalisation and mortality in patients with a registered diagnosis of chronic kidney disease (CKD), with and without type 2 diabetes. **(A)** The broad outcome definition used diagnoses in any discharge position and **(B)** the strict outcome definition used diagnoses in first position only. The rates of events are presented as the number of events per 100 patient-years and only includes diagnoses made in inpatient care

Adverse outcomes were monitored in 120,549 patients with diagnosed CKD (Table S1). Most adverse outcomes were more common in patients with CKD and T2D than those without T2D: rates of hospitalisation for CKD (28.1 *versus* 22.1 events per 100 patient years, respectively), heart failure (12.6 *versus* 9.8), myocardial infarction (3.9 vs. 2.2), stroke (3.2 vs. 2.3). However, mortality (8.5 vs. 10.8 events per 100 patient years) was slightly higher in patients without T2D. Risks were mainly driven by cardiorenal disease events (CKD and/or heart failure hospitalisations). Event rates for cardiorenal disease and ASCVD were slightly higher in patients with T2D than those without T2D (Fig. 1 and Table S2). The same trends were observed when using either a broad or strict outcome definition. Mortality rates were similar between patients with T2D and patients without T2D up until the age of 75 years. Thereafter, patients with T2D had a higher risk of mortality (Figure S1).

### Hospital health care costs

**Fig. 2:**
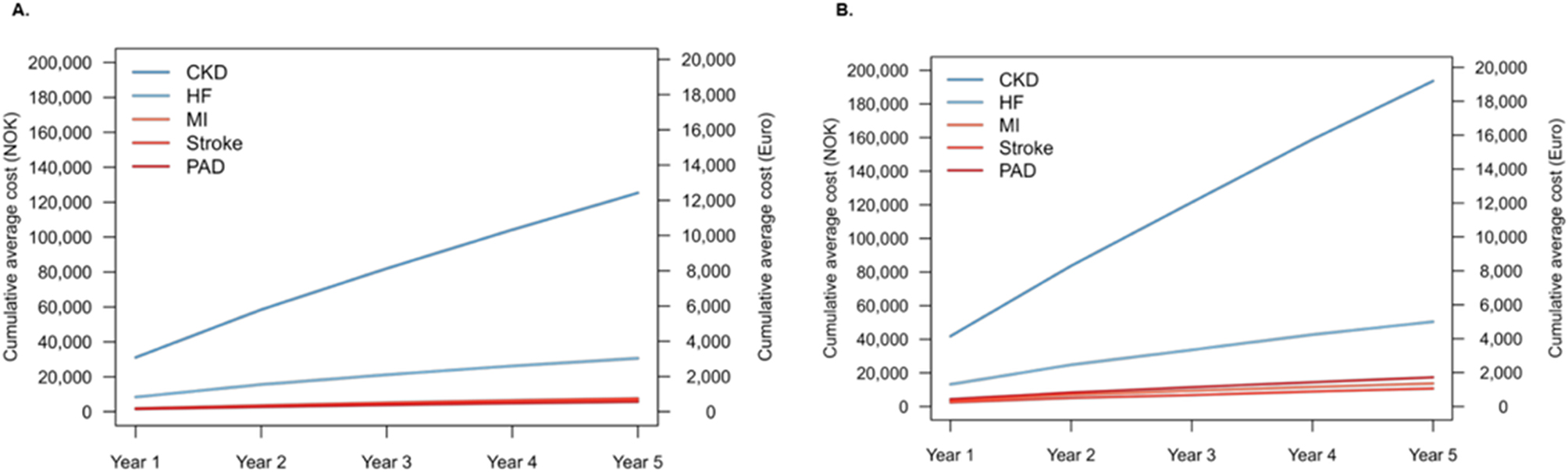
The average cumulative cost of outpatient and inpatient care per patient with a registered diagnosis of chronic kidney disease, without **(A)** and with **(B)** type 2 diabetes, each year from and including 2018 to 2022. T2D denotes type 2 diabetes; NOK, Norwegian Kronor (currency); CKD, chronic kidney disease; HF, heart failure; MI, myocardial infarction; PAD, peripheral artery disease

Health care costs were monitored in 97,017 patients with diagnosed CKD (Table S3). Each year the costs of CKD and heart failure were consistently higher than those for ASCVD, with annual costs generally higher in patients with T2D (Fig. 2). After five years follow-up, the total cost of care for cardiorenal events was approximately five times higher than that for ASCVD. Total costs were approximately 50% higher for patients with T2D than those without T2D.

### The uptake of SGLT-2 inhibitors

**Fig. 3:**
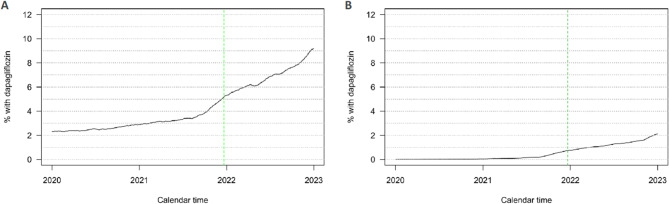
The proportions of patients with a registered diagnosis of chronic kidney disease, **(A) **with and **(B)** without type 2 diabetes, who were treated with the sodium-glucose cotransporter-2 inhibitor, dapagliflozin, each day on and between January 1st, 2020, and December 31st, 2022. The dotted green line indicates the date on which dapagliflozin was approved in Norway (December 21st, 2021) for use in treating chronic kidney disease, regardless of whether the patient has type 2 diabetes or not

The utilisation of the SGLT-2 inhibitor, dapagliflozin, increased approximately two-fold in the calendar year after its approval for CKD treatment (i.e., from the beginning to the end of 2022; Fig. 3); from 5.3 to 9.3% and from 1.0 to 2.1% in patients with T2D and patients without T2D, respectively.

### Medication utilisation in new users of SGLT-2 inhibitors and RAS inhibitors

**Fig. 4:**
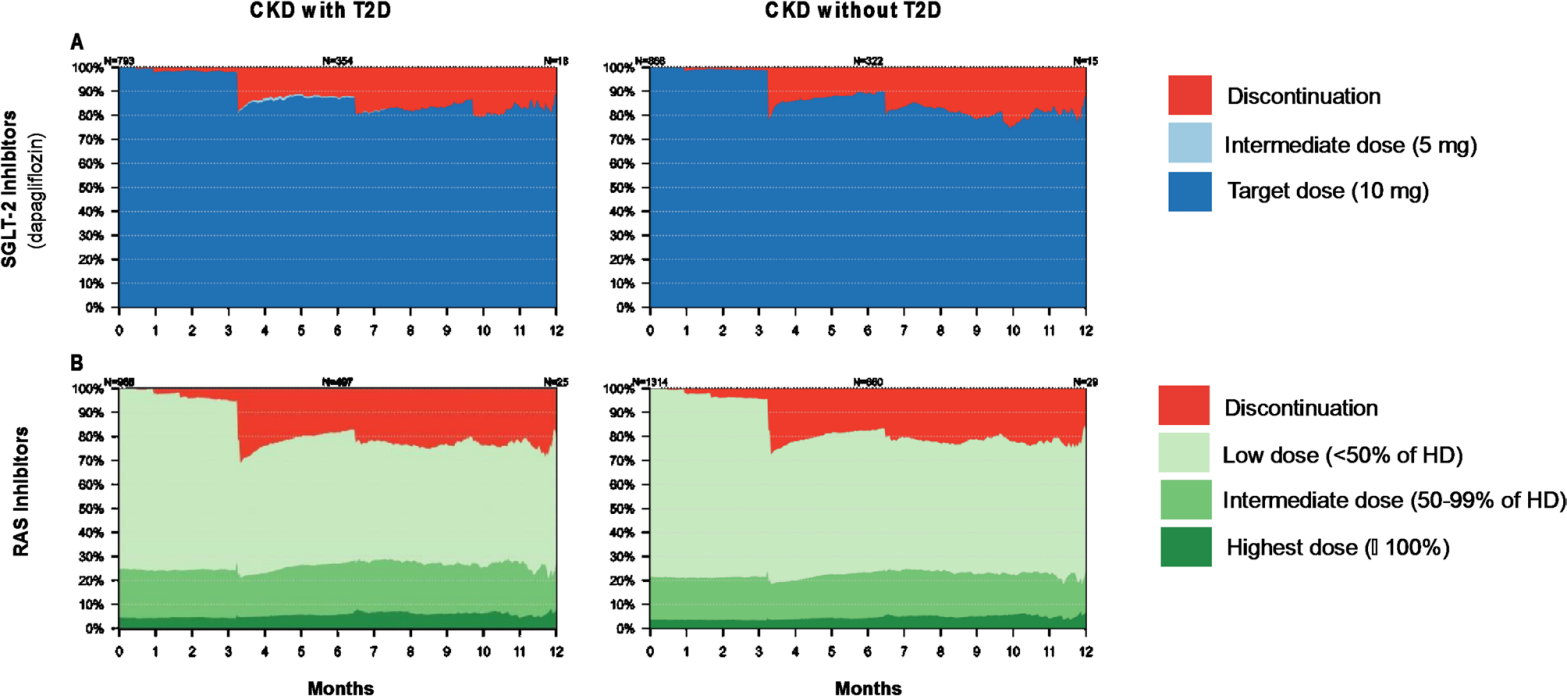
Persistence with **(A)** SGLT-2 inhibitor (dapagliflozin) and **(B)** RAS inhibitor treatment in patients with chronic kidney disease, with and without type 2 diabetes, who were new users of the medications. CKD denotes chronic kidney disease; T2D, type 2 diabetes; SGLT-2, sodium-glucose transporter-2; RAS, renin-angiotensin system

Following the approval of the SGLT-2 inhibitor, dapagliflozin, for CKD treatment, 1,659 and 1,760 patients with CKD initiated treatment with dapagliflozin and/or RAS inhibitors, respectively (Table S3). Following new initiation, less than 20% of patients who used dapagliflozin discontinued their treatment and almost all these patients maintained their target dose of 10 mg throughout 12-months follow-up, irrespective of diabetes status (Fig. 4). New initiation of RAS inhibitor treatment was followed by slightly higher discontinuation rates, with the medication prescribed mainly at a low dose.

## Discussion

In this nationwide study, the estimated prevalence of diagnosed CKD increased by 18% in Norway over the three years prior to 2023. Over two thirds (73%) of these patients did not have T2D. Comorbidities, risk of severe complications, and mortality were substantial independently of diabetes status. The utilisation of kidney-protective medications such as SGLT-2 inhibitors and RAS inhibitors was low, particularly in patients without T2D. Hospital health care costs were mainly driven by cardiorenal complications and were generally higher in patients with T2D. Novel kidney-protective treatment with the SGLT-2 inhibitor, dapagliflozin, increased two-fold in the year after its approval for CKD treatment, irrespective of diabetes status. Dapagliflozin was almost always used at its target dose, while RAS inhibitor treatment was mostly used at a low dose, the latter associated with higher discontinuation rates.

### Prevalence and characteristics

When comparing with a previous report that also reported Norwegian data [5], the prevalence of diagnosed CKD in Norway appears to have increased by 18% over the three years prior to the beginning of 2023. This increase in prevalence is likely explained by improved awareness and subsequent increased diagnosis rates, potentially resulting from the paradigm shifting efficacy results from the DAPA-CKD trial in 2020 [1], followed by the approval of dapagliflozin for the treatment of CKD in 2021; i.e., both reported efficacy and the availability of novel kidney-protective medications. The prevalence of T2D among patients with CKD supports findings in other recent studies [2–5].

### Risks

Patients who had a combination of CKD and T2D had more comorbidity than those without T2D, similar to patients in other countries [3]. Despite differences in the burden of comorbidities, the risk of hospitalisation for cardiorenal and ASCVD, and the risk of mortality were relatively similar between each cohort, suggesting that T2D might not fully explain the high risk of adverse outcomes in CKD. Indeed, most of the patients with and without T2D in this study have stage 3–5 CKD and, thus, long standing hypertension and a history of poor cardiovascular disease risk management, establishing advanced profiles of cardiovascular and cardiorenal risk that may lessen the prominence of the risk associated with T2D [5]. Additionally, the high prevalence of stage 3–5 CKD in this study provides an initial indication of how severely underdiagnosed CKD is and how undertreated in its early stages (i.e., stage 1–2 CKD). Patients with CKD and T2D over 75 years of age had a higher risk of adverse outcomes than those without T2D. However, given that this study did not analyse if the characteristics of patients with diagnosed CKD vary by age group, this study cannot provide an explanation of why risk is elevated in older patients.

### Health care costs

Although the risk of hospitalisation was similar between each cohort, the costs of health care associated with cardiorenal and ASCVD outcomes were 50% higher for patients with T2D; still acknowledging that health care costs were also substantial for patients without T2D. The reason for the difference in cost between patients with and without T2D was not elucidated in this study. Consistent with recent data from North America, other European nations, and Japan [3, 5], the costs associated with hospitalisation for cardiorenal disease were substantially higher than those for ASCVD in patients with CKD, regardless of whether they had T2D or not.

### Underutilisation of kidney-protective treatment

In addition to its antihyperglycemic effect, treatment with a SGLT-2 inhibitor and/or RAS inhibitor provides many benefits to patients with CKD, including reduced albuminuria and proteinuria, delayed CKD progression, and prevention of renal and cardiovascular events and death [1, 8]. Despite the similarities in the risks of severe morbidity and mortality between patients with and patients without T2D, there was a pronounced underutilisation of novel kidney-protective treatments at baseline in patients without T2D, those who made up most of the population in this study. This has been demonstrated before in patients with incident stage 3–4 CKD [3]. The difference may partly be explained by patients with T2D who initiated SGLT2 inhibitor treatment for its glucose lowering effect. Additionally, there may be differences in adherence to treatment recommendations and structured follow-up visits between patients with and those without T2D.

### Uptake of SGLT-2 inhibitors

The two-fold increase in the utilisation of the SGLT-2 inhibitor, dapagliflozin, by patients with and without T2D is a promising development. However, the absolute uptake of dapagliflozin was still low. This might be explained by the gap between the approval of dapagliflozin for the treatment of CKD and its reimbursement, which came 11 months later in November 2022 toward the end of the follow-up period. In comparison to Japan, Finland, the United States, and Sweden, uptake seemed slightly higher in Norway [3, 9].

Dapagliflozin was almost always utilized at its highest target dose, 10 mg, and rates of discontinuation were lower than that with RAS inhibitors. The latter were mostly administered at low doses with little sign of up-titration. The extensive use of RAS inhibitors at a low dose might be explained by the lack of a target dose for the medication in CKD treatment guidelines, the risk of side effects e.g. hyperkalemia, and that RAS inhibitor treatment is often initiated at a low dose with an aim of up-titration [3]. This is in contrast to the heart failure treatment guidelines where target doses are clearly defined [3]. The effects of submaximal dosing and discontinuation of RAS inhibitors on the risk of adverse outcomes has not yet been reported.

### Strengths and limitations

This study had access to health registries with records of most hospital health care and medication dispensations, and with mandatory reporting of all causes of death, for all adult residents of Norway. Additionally, the costs of hospital health care for cardiorenal diseases and ASCVD reflect the actual cost charged by the healthcare provider. Despite its strength in its capacity to characterise the comorbidities, risks, associated hospital health care costs, and treatments in a contemporary patient population with diagnosed CKD, several limitations must be acknowledged.

This study did not have access to laboratory data, limiting the clinical characterisation of the patients. Further, without access to measures of glomerular filtration rate, patients with CKD were identified based only on diagnoses recorded in the Norwegian Patient Register. Given that many patients in similar healthcare settings have CKD according to measurements of glomerular filtration rate, yet remain undiagnosed by healthcare [2], and that records of CKD diagnoses in primary care were not available, it seems obvious that this study underestimates the prevalence of CKD in Norway.

Finally, this study did not assess if characteristics or treatments differ by gender, ethnicity, or socioeconomic background, limiting the generalizability of this study’s finding. However, similarities between this study’s findings and those from other evaluations of patients with CKD suggest that the results may be generalizable to other countries with similar healthcare settings.

## Conclusion

This study demonstrates that the prevalence of diagnosed CKD has increased by 18% over the last three years and that most patients with established CKD do not have diabetes. A large proportion of patients were not treated with RAS inhibitors or SGLT-2 inhibitors as recommended in CKD treatment guidelines. The proportion not treated was larger in patients without T2D. Risks and health care costs were high and mainly driven by cardiorenal causes, irrespective of diabetes status; highlighting a need for improved risk management and treatment initiation. While the uptake of SGLT-2 inhibitors in CKD has increased substantially, their utilisation remains generally low. Although this study is limited by its lack of access to laboratory measures of CKD and a short period of observation of SGLT-2 inhibitor utilisation following its approval for full-reimbursement, these results highlight an urgent need for improved kidney-protective treatment to improve patient outcomes in CKD.

## Electronic supplementary material

Below is the link to the electronic supplementary material.

Supplementary Material 1

Supplementary Material 2

## Data Availability

Data sources utilised in this project are available on reasonable request to the corresponding author.
