# Supplementary material for: Prevalence, outcomes, costs, and treatments of a contemporary population with chronic kidney disease in Norway: a nationwide observational study
Source: BMC Nephrol. 2025 Jul 17;26:393. doi: 10.1186/s12882-025-04171-7 (PMC12273365; doi:10.1186/s12882-025-04171-7)
Supplement: Supplementary file 1 — Supplementary Material 1 [file 12882_2025_4171_MOESM1_ESM.docx]

Supplemental Material

Contents

[Supplemental Methods 1 2](#_Toc184631639)

[Supplemental Methods 2 3](#_Toc184631640)

[Supplemental Methods 3 4](#_Toc184631641)

[Supplemental Methods 4 5](#_Toc184631642)

[Supplemental Methods 5 6](#_Toc184631643)

[Supplemental Methods 6 7](#_Toc184631644)

[Table S1. 8](#_Toc184631645)

[Table S2. 10](#_Toc184631646)

[Table S3. 11](#_Toc184631647)

[Table S4. 13](#_Toc184631648)

[References 15](#_Toc184631649)

Supplemental Methods 1

Data were extracted from Norway’s Patient Register, Prescription Database, and Cause of Death Register. The Norwegian Patient Register contains records of all diagnoses and surgical procedures made during visits to outpatient and inpatient clinics in Norway since 2008, with diagnoses recorded according to the International Classification of Disease-10 (ICD-10) coding system (1), and surgical procedures recorded according to the Nordic Medico-Statistical Committee Classification of Surgical Procedure system (2). The reporting of the cause of death is mandatory in Norway and all deaths that occurred since 1958 are recorded in Norway’s Cause of Death Register according to the ICD-10 system. The Norwegian Prescription Database contains records of all filled prescriptions in Norway, with the type of medication that was dispensed recorded according to the Anatomical Therapeutic Chemical (ATC) Classification system (3).

Supplemental Methods 2

The following ICD-10 and procedure codes were used to identify patients with chronic kidney disease: N00-N08, N10-N19, I12.0-I12.9, I13.1, I13.2, E10.2, E11.2, E12.2, E13.2, E14.2, Z49, Z99.2, codes for dialysis and kidney transplantation: JAK10, TJA20, TJA33, DJ008, QF006, DR015- DR024.

# Supplemental Methods 3

Patients with type 2 diabetes were identified if they had record of treatment with a glucose-lowering drug during the year prior to the index date (ATC code, A10). Those on monotherapy with a sodium-glucose cotransporter-2 inhibitor were not considered to have type 2 diabetes.

# Supplemental Methods 4

The following ICD-10 and procedure codes were used to identify comorbidities in patients with a registered diagnosis of chronic kidney disease, with or without type 2 diabetes:

| **Comorbidity** | **ICD-10 code** | **Other** |
| --- | --- | --- |
| Myocardial infarction | I21-I22, I25.2, I25.6 |  |
| Unstable angina | I20.0 |  |
| Angina pectoris | I20.1, I20.8, I20.9, I25.1, I25.5 | ATC: C01DA |
| Heart failure | I50, I11.0, I13.0, I13.2 |  |
| Stroke | I60-I66, G45 |  |
| Atrial fibrillation | I48 |  |
| Peripheral artery disease | I70.2, I73.9, I74.2-9 |  |
| Hyperkalemia | E875 |  |
| Cancer | C00-C99 |  |

# Supplemental Methods 5

The following ATC codes were used to identify use of medications of interest by each patient with a registered diagnosis of chronic kidney disease, with or without type 2 diabetes:

| **Medication** | **ATC codes** |
| --- | --- |
| SGLT-2 inhibitors | A10BK, A10BD15, A10BD16, A10BD20 |
| Dapagliflozin | A10BK01, A10BD15 |
| RAS inhibitors (ACEi/ARB/ARNi) | C09A, C09B, C09C, C09D (excluding C09DX04) |
| MRA | C03DA |
| Statins | C10AA |
| Loop-diuretics | C03C |
| Low-dose aspirin | B01AC06 |
| Potassium binders | V03AE01, V03AE09, V03AE10 |
| Metformin | A10BA |
| Sulphonylureas | A10BB |
| DPP-4 inhibitors | A10BH |
| GLP-1 agonists | A10BJ |
| Insulin | A10A |

RAS denotes renin-angiotensin system; ACEi, angiotensin-converting enzyme inhibitors; ARB, angiotensin receptor blockers; ARNi, angiotensin receptor-neprilysin inhibitors; SGLT-2, sodium-glucose cotransporter 2; DPP-4, dipeptidyl peptidase-4; glucagon-like peptide-1.

# Supplemental Methods 6

The doses for each type of renin-angiotensin system inhibitor used to categorise them as low, medium, or high dose are listed below:

| **RAS inhibitors** |  | | |
| --- | --- | --- | --- |
|  | **Low (mg)** | **Intermediate (mg)** | **High (mg)** |
| **ACE inhibitors** |  |  |  |
| Captopril | N/A | 25 | 50 |
| Enalapril | 2.5–5 | 10 | 20 |
| Lisinopril | 5 | 10 | 20 |
| Perindopril | 2 | 4 | 8 |
| Ramipril | 1.25–2.5 | 10 | 20 |
| **ARB** |  |  |  |
| Candesartan | 4–8 | 16 | 32 |
| Irbesartan | 75 | 150 | 300 |
| Losartan | 12.5–25 | 50 | 100 |
| Telmisartan | 20 | 40 | 80 |
| Valsartan | 40–80 | 160 | 320 |

RAS denotes renin-angiotensin system; ACEi, angiotensin-converting enzyme inhibitors; ARB, angiotensin receptor blockers.

Table S1. The characteristics of patients in Norway with a registered diagnosis of chronic kidney disease as of January 1^st^, 2022, the cohort in which adverse events were monitored

|  | **All patients with CKD*** | **CKD with T2D** | **CKD without T2D** | **Standardised difference (%)** |
| --- | --- | --- | --- | --- |
| **n** | 120,549 | 29,241 | 90,176 |  |
| **Age, years (SD)** | 70 (16) | 70 (13) | 70 (17) | 0.0 |
| **Female, n (%)** | 50,281 (42) | 11,373 (39) | 38,366 (43) | 7.4 |
| **Kidney disease diagnoses, n (%)** |  |  |  |  |
| Chronic | 56,618 (52) | 13,080 (54) | 43,209 (52) | 5.3 |
| Stage 1-2 | 6,464 (14) | 1,039 (9) | 5,384 (15) | 18.7 |
| Stage 3-4 | 33,038 (71) | 8,351 (74) | 24,526 (70) | 9.3 |
| Stage 5 | 7,251 (16) | 1,906 (17) | 5,245 (15) | 5.3 |
| Acute kidney injury | 55,921 (51) | 11,058 (46) | 44,557 (53) | 14.8 |
| Unspecified | 22,011 (20) | 5,132 (21) | 16,783 (20) | 3.0 |
| Diabetic | 9,918 (9) | 7,968 (33) | 1,260 (2) | 91.7 |
| Hypertensive | 6,616 (6) | 1,590 (7) | 4,994 (6) | 2.6 |
| Glomerular diseases | 8,654 (7) | 2,041 (7) | 6,455 (7) | 0.7 |
| Renal tubulo-interstitial diseases | 27,177 (23) | 8,246 (28) | 18,680 (21) | 17.5 |
| Dialysis | 6,121 (6) | 1,493 (6) | 4,550 (5) | 3.2 |
| **Comorbidities, n (%)** |  |  |  |  |
| Cardiovascular disease | 63,435 (53) | 17,090 (58) | 46,013 (51) | 14.9 |
| Myocardial infarction | 22,371 (19) | 6,877 (24) | 15,373 (17) | 16.1 |
| Unstable angina | 6,494 (5) | 2,241 (8) | 4,208 (5) | 12.5 |
| Angina pectoris | 31,343 (26) | 9,954 (34) | 21,182 (23) | 23.5 |
| Heart failure | 26,049 (22) | 6,603 (23) | 19,359 (21) | 2.7 |
| Stroke | 13,490 (11) | 3,614 (12) | 9,821 (11) | 4.6 |
| Atrial Fibrillation | 30,953 (26) | 7,223 (25) | 23,672 (26) | 9.9 |
| Peripheral artery disease | 11,377 (9) | 3,705 (13) | 7,548 (8) | 3.6 |
| Microvascular complications | 28,773 (24) | 19,913 (68) | 7,791 (9) | 154.5 |
| Severe hyperkalemia | 7,814 (6) | 2,366 (8) | 5,352 (6) | 8.5 |
| Obesity | 10,320 (9) | 5,066 (17) | 5,130 (6) | 37.1 |
| Cancer | 36,254 (30) | 8,286 (28) | 27,871 (31) | 5.6 |
| **Medications, n (%)** |  |  |  |  |
| SGLT-2 inhibitors | 6,992 (6) | 5,478 (19) | 1,482 (2) | 58.9 |
| RAS inhibitors | 61,477 (51) | 18,973 (65) | 41,910 (46) | 37.7 |
| MRA | 7,677 (6) | 2,372 (8) | 5,271 (6) | 8.9 |
| Statins | 58,721 (49) | 20,764 (71) | 37,306 (41) | 62.6 |
| Loop-diuretics | 30,473 (25) | 9,639 (33) | 20,650 (23) | 22.6 |
| Low-dose aspirin | 35,944 (30) | 11,814 (40) | 23,829 (26) | 30.0 |
| Potassium binders | 1,826 (2) | 622 (2) | 1,176 (1) | 6.3 |
| **Diabetes medication, n (%)** |  |  |  |  |
| Metformin | 14,386 (12) | 14,338 (49) | 0 (0) | 138.7 |
| Sulphonylureas | 2,441 (2) | 2,438 (8) | 0 (0) | 42.7 |
| DPP-4 inhibitors | 7,531 (6) | 7,515 (26) | 0 (0) | 83.2 |
| GLP-1 agonists | 7,349 (6) | 7,311 (25) | 0 (0) | 81.7 |
| Insulin | 15,432 (13) | 14,312 (49) | 0 (0) | 138.5 |

CKD denotes chronic kidney disease; T2D, type 2 diabetes; SD, standard deviation; RAS, renin-angiotensin system; MRA, mineral corticoid antagonists; SGLT-2, sodium glucose transport-2; DPP-4, Dipeptidyl peptidase-4; GLP-1, glucagon-like peptide-1. Standardised differences >10% indicate non-negligible differences.

Table S2. One-year event rates (events per 100 patient-years) of hospitalisation and mortality in patients with a registered diagnosis of chronic kidney disease, with and without type 2 diabetes

|  | **Outpatient/inpatient diagnoses** | | **Inpatient diagnoses** | |
| --- | --- | --- | --- | --- |
| **Event** | **CKD with T2D**  **n=27,717** | **CKD without T2D**  **n=90,176** | **CKD with T2D**  **n=26,049** | **CKD without T2D**  **n=84,477** |
| All-cause death | 2215 (8.3) | 9168 (10.8) | 2215 (8.3) | 9168 (10.8) |
| CV death | 599 (2.3) | 2552 (3.0) | 599 (2.3) | 2552 (3.0) |
| Renal death | 141 (0.5) | 490 (0.6) | 141 (0.5) | 490 (0.6) |
| Cardiorenal disease | 8118 (35.3) | 19943 (25.9) | 3109 (11.8) | 7478 (8.8) |
| Heart failure | 2772 (10.7) | 6815 (8.2) | 1592 (6.0) | 3707 (4.4) |
| CKD | 6342 (26.8) | 14660 (18.6) | 1798 (6.8) | 4249 (5.0) |
| MI | 803 (3.0) | 1432 (1.7) | 736 (2.8) | 1323 (1.6) |
| Stroke | 839 (3.2) | 1993 (2.3) | 715 (2.7) | 1678 (2.0) |
| PAD | 1221 (4.6) | 1978 (2.3) | 529 (2.0) | 720 (0.8) |
| Any hospitalisation | - | - | 8359 (37.4) | 25039 (34.3) |

Cardiorenal disease is a combination of CKD and/or heart failure. CV denotes cardiovascular; CKD, chronic kidney disease; MI, myocardial infarction; PAD, peripheral artery disease.

Table S3. The characteristics of patients in Norway with a registered diagnosis of chronic kidney disease as of January 1^st^, 2018, the sub-cohort in which hospital health care costs were monitored

|  | **All patients with CKD*** | **CKD with T2D** | **CKD without T2D** | **Standardised difference (%)** |
| --- | --- | --- | --- | --- |
| **n** | 97,017 | 21,049 | 75,045 |  |
| **Age, years (SD)** | 70 (16) | 70 (13) | 70 (17) | 3.1 |
| **Female, n (%)** | 41061 (42) | 8152 (39) | 32465 (43) | 9.2 |
| **Kidney disease diagnoses, n (%)** |  |  |  |  |
| Chronic | 47621 (55) | 10120 (56) | 37192 (55) | 1.6 |
| Stage 1-2 | 4564 (13) | 692 (8) | 3851 (14) | 17.1 |
| Stage 3-4 | 25766 (71) | 6061 (74) | 19547 (70) | 9.0 |
| Stage 5 | 5877 (16) | 1396 (17) | 4382 (16) | 3.7 |
| Acute kidney injury | 38883 (45) | 7034 (39) | 31633 (47) | 16.3 |
| Unspecified | 18066 (21) | 3856 (21) | 14127 (21) | 0.9 |
| Diabetic | 8171 (9) | 6579 (36) | 1011 (1) | 99.2 |
| Hypertensive | 6172 (7) | 1387 (8) | 4748 (7) | 2.4 |
| Glomerular diseases | 7370 (8) | 1531 (7) | 5681 (8) | 1.1 |
| Renal tubulo-interstitial diseases | 20351 (21) | 4728 (22) | 15422 (21) | 4.7 |
| Dialysis | 5150 (6) | 1124 (6) | 3939 (6) | 1.6 |
| **Comorbidities, n (%)** |  |  |  |  |
| Cardiovascular disease | 50221 (52) | 12462 (59) | 37493 (50) | 18.6 |
| Myocardial infarction | 18627 (19) | 5323 (25) | 13210 (18) | 18.8 |
| Unstable angina | 4962 (5) | 1582 (8) | 3349 (4) | 12.9 |
| Angina pectoris | 24105 (25) | 7045 (33) | 16885 (22) | 24.6 |
| Heart failure | 20162 (21) | 5013 (24) | 15073 (20) | 9.0 |
| Stroke | 10107 (10) | 2494 (12) | 7568 (10) | 5.6 |
| Atrial Fibrillation | 23888 (25) | 5242 (25) | 18607 (25) | 0.3 |
| Peripheral artery disease | 8857 (9) | 2743 (13) | 6009 (8) | 16.4 |
| Microvascular complications | 21368 (22) | 15067 (72) | 5436 (7) | 174.9 |
| Severe hyperkalemia | 5219 (5) | 1519 (7) | 3620 (5) | 10.1 |
| Obesity | 6231 (6) | 2852 (14) | 3302 (4) | 32.4 |
| Cancer | 25530 (26) | 5005 (24) | 20459 (27) | 8.0 |
| **Medications, n (%)** |  |  |  |  |
| SGLT-2 inhibitors | 1841 (2) | 1683 (8) | 148 (0) | 40.1 |
| RAS inhibitors | 49142 (51) | 14067 (67) | 34600 (46) | 42.7 |
| MRA | 5210 (5) | 1647 (8) | 3534 (5) | 12.9 |
| Statins | 45074 (46) | 14697 (70) | 29903 (40) | 63.2 |
| Loop-diuretics | 27590 (28) | 8310 (39) | 19074 (25) | 30.4 |
| Low-dose aspirin | 33984 (35) | 10202 (48) | 23494 (31) | 35.6 |
| Potassium binders | 792 (1) | 203 (1) | 570 (1) | 2.2 |
| **Diabetes medication, n (%)** |  |  |  |  |
| Metformin | 9853 (10) | 9811 (47) | 0 (0) | 132.1 |
| Sulphonylureas | 3650 (4) | 3648 (17) | 0 (0) | 64.8 |
| DPP-4 inhibitors | 6337 (7) | 6322 (30) | 0 (0) | 92.7 |
| GLP-1 agonists | 1826 (2) | 1819 (9) | 0 (0) | 43.5 |
| Insulin | 11980 (12) | 11064 (53) | 0 (0) | 148.9 |

CKD denotes chronic kidney disease; T2D, type 2 diabetes; SD, standard deviation; RAS, renin-angiotensin system; MRA, mineral corticoid antagonists; SGLT-2, sodium glucose transport-2; DPP-4, Dipeptidyl peptidase-4; GLP-1, glucagon-like peptide-1. Standardised differences >10% indicate non-negligible differences.

Table S4. The characteristics of patients in Norway with a registered diagnosis of chronic kidney disease who were new users of dapagliflozin and/or renin-angiotensin system inhibitors after dapagliflozins approval in Norway for use in all patients with chronic kidney disease

|  | **Dapagliflozin** | **RAS inhibitors** |
| --- | --- | --- |
| **n** | 1,659 | 1,760 |
| **Age, years (SD)** | 70 (13) | 67 (18) |
| **Female, n (%)** | 462 (28) | 642 (36) |
| **Days since first CKD diagnosis, median (IQR)** | 818 (88-2323) | 278 (24-1897) |
| **Days since last CKD diagnosis, median (IQR)** | 14 (4-73) | 18 (5-99) |
| **Any inpatient hospitalization with CKD in 2021** | 1028 (62) | 1201 (68) |
| **Comorbidities, n (%)** |  |  |
| Any heart failure | 809 (49) | 461 (26) |
| Ischemic heart disease | 295 (18) | 217 (12) |
| Myocardial infarction | 291 (18) | 212 (12) |
| Kidney disease | 1659 (100) | 1760 (100) |
| Diabetes | 793 (48) | 446 (25) |
| Atrial fibrillation | 497 (30) | 347 (20) |
| Stroke total | 77 (5) | 117 (7) |
| Peripheral arterial disease | 75 (5) | 59 (3) |
| Cancer | 204 (12) | 269 (15) |
| Covid-19 infection | 0 (0) | 0 (0) |
| **Medication history, n (%)** |  |  |
| RAAS inhibitors | 1405 (85) | 0 (0) |
| Beta blockers | 1152 (69) | 888 (50) |
| MRA | 457 (28) | 179 (10) |
| ARNI | 181 (11) | 0 (0) |
| SGLT-2 | 0 (0) | 234 (13) |
| GLP-1 | 228 (14) | 99 (6) |
| DPP-4 | 310 (19) | 141 (8) |
| High ceiling diuretics | 910 (55) | 631 (36) |
| Ivabradine | 4 (0) | 2 (0) |
| Nitrates | 210 (13) | 145 (8) |
| Vitamin K antagonists | 102 (6) | 76 (4) |
| Receptor P2Y12 antagonists | 266 (16) | 237 (13) |
| Potassium binders | 58 (3) | 33 (2) |
| Pacemaker or defibrillator | 12 (1) | 7 (0) |

CKD denotes chronic kidney disease; RAS, renin-angiotensin system; SD, standard deviation; IQR, interquartile range; RAAS, renin-angiotensin aldosterone system; MRA, mineral corticoid antagonists; ARNI, angiotensin receptor-neprilysin inhibitors; SGLT-2, sodium glucose transport-2; GLP-1, glucagon-like-peptide-1; DPP-4, Dipeptidyl peptidase-4.


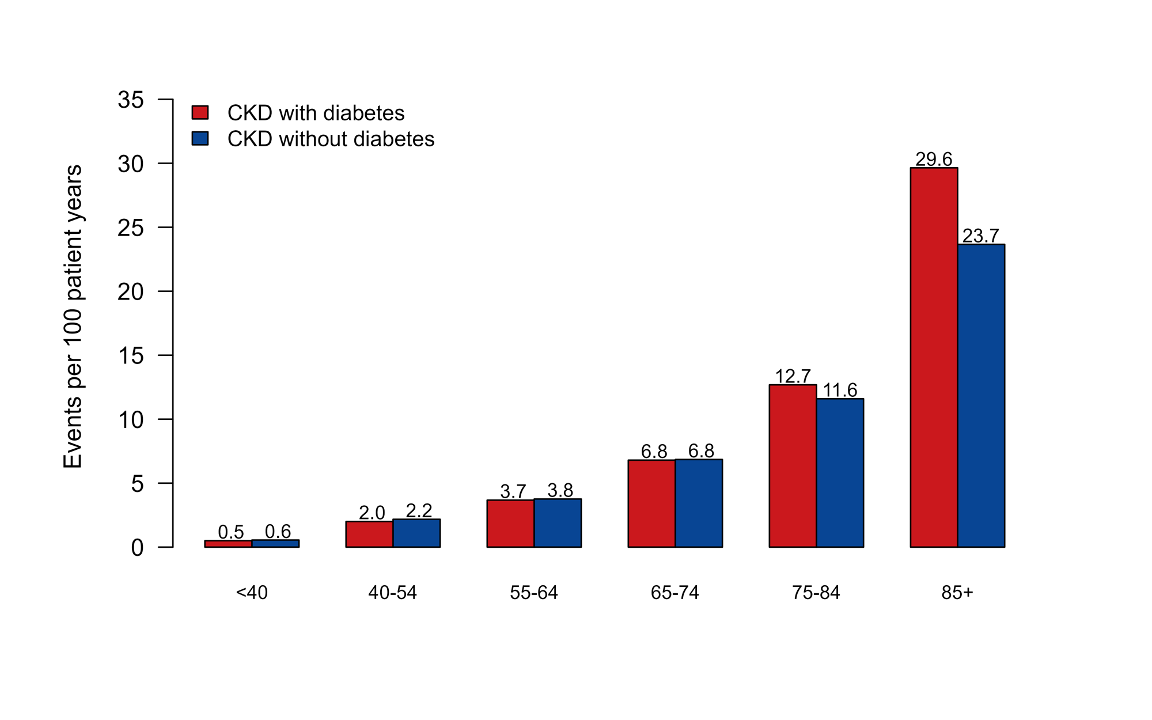
Figure S1. **One-year event rates of mortality in patients with a registered diagnosis of chronic kidney disease (CKD), with and without type 2 diabetes, stratified by age groups.** The rates of events are presented as the number of events per 100 patient-years.

References

1. World Health Organization. International classification of disease and related health problems. Available from: http://www.who.int/classifications/icd/en/. Accessed September 21^st^, 2024.

2. Nordic Medico-Statistical Committee. NOMESCO Classification of Surgical Procedures. 2011. Available from: https://norden.diva-portal.org/smash/get/diva2:968721/FULLTEXT01.pdf. Accessed September 21^st^, 2024.

3. World Health Organization. WHO Collaborating Centre for Drug Statistics Methodology. ATC/DDD Index. 2024. Available from: https://www.whocc.no/. Accessed September 21^st^, 2024.
