# Supplementary figures and images for: Prevalence, outcomes, costs, and treatments of a contemporary population with chronic kidney disease in Norway: a nationwide observational study
Source: BMC Nephrol. 2025 Jul 17;26:393. doi: 10.1186/s12882-025-04171-7 (PMC12273365; doi:10.1186/s12882-025-04171-7)

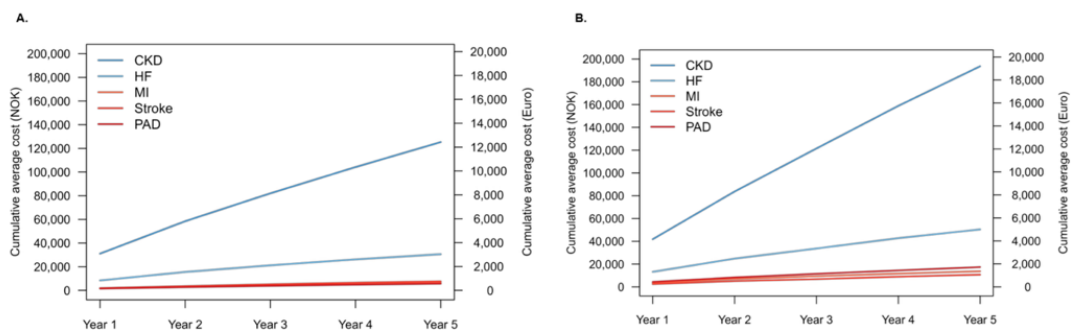

**Figure 2**

Supplement: Supplementary file 2 — Supplementary Material 2 [file 12882_2025_4171_MOESM2_ESM.pdf]
